# SCAN database: facilitating integrative analyses of cytosine modification and expression QTL

**DOI:** 10.1093/database/bav025

**Published:** 2015-03-26

**Authors:** Wei Zhang, Eric R. Gamazon, Xu Zhang, Anuar Konkashbaev, Cong Liu, Keely L. Szilágyi, M. Eileen Dolan, Nancy J. Cox

**Affiliations:** ^1^Department of Preventive Medicine, Northwestern University Feinberg School of Medicine, Chicago, IL 60611, USA, ^2^The Affiliated Hospital of Medical School, Ningbo University, Ningbo, Zhejiang Province, China, ^3^Section of Genetic Medicine, Department of Medicine, University of Chicago, Chicago, IL 60637, USA, ^4^Section of Hematology/Oncology, Department of Medicine, University of Illinois at Chicago, Chicago, IL 60612, USA, ^5^Department of Bioengineering, University of Illinois at Chicago, Chicago, IL 60612, USA, ^6^Biological Resources Laboratory, University of Illinois at Chicago, Chicago, IL 60612, USA and ^7^Section of Hematology/Oncology, Department of Medicine, University of Chicago, Chicago, IL 60637, USA

## Abstract

Functional annotation of genetic variants including single nucleotide polymorphisms (SNPs) and copy number variations (CNV) promises to greatly improve our understanding of human complex traits. Previous transcriptomic studies involving individuals from different global populations have investigated the genetic architecture of gene expression variation by mapping expression quantitative trait loci (eQTL). Functional interpretation of genome-wide association studies (GWAS) has identified enrichment of eQTL in top signals from GWAS of human complex traits. The SCAN (SNP and CNV Annotation) database was developed as a web-based resource of genetical genomic studies including eQTL detected in the HapMap lymphoblastoid cell line samples derived from apparently healthy individuals of European and African ancestry. Considering the critical roles of epigenetic gene regulation, cytosine modification quantitative trait loci (mQTL) are expected to add a crucial layer of annotation to existing functional genomic information. Here, we describe the new features of the SCAN database that integrate comprehensive mQTL mapping results generated in the HapMap CEU (Caucasian residents from Utah, USA) and YRI (Yoruba people from Ibadan, Nigeria) LCL samples and demonstrate the utility of the enhanced functional annotation system.

**Database URL:** http://www.scandb.org/

## Introduction

High-throughput genotyping and sequencing technologies have facilitated genome-wide scans of genetic variants associated with human complex traits including quantitative traits and risks for common, complex diseases. To date, genome-wide association studies (GWAS) have identified genetic variants, particularly single nucleotide polymorphisms (SNPs), associated with more than 500 traits (1). For example, the National Human Genome Research Institute (NHGRI) GWAS Catalog (1) has curated a list of >15 000 SNPs associated with more than 500 traits from the ever-increasing number of GWAS publications. Except for the rare instances in which a GWAS locus is already known to affect certain biological functions, the majority of the identified GWAS loci remain to be functionally characterized. Understanding the functional basis for genetic variants associated with human complex traits is, therefore, critical for understanding the underlying biological processes.

Intermediate molecular phenotypes (e.g. gene expression and cytosine modification) are clearly defined traits with a strong genetic component. Previous studies using the International HapMap Project (2, 3) human lymphoblastoid cell line (LCL) samples have identified expression quantitative trait loci (eQTL), particularly *cis*-acting or local eQTL implicated in gene regulation (4, 5). GWAS of complex trait-associated loci (6) as well as toxicity-associated loci detected for various anticancer chemotherapies (7) are enriched in eQTL, thus providing important novel annotations for these genetic variants and contributing to an improved understanding of their functional consequences. The SNP and Copy Number Variant (CNV) Annotation (SCAN) database (8) was developed specifically to store and serve the eQTL mapping data identified using this widely used human genetics model, and has been welcomed by the research community as evidenced by >2.5 million unique queries from >56 000 unique IP addresses since its launch in 2009 (09/2009–10/2014). Applications of SCAN may include prioritization and annotation of GWAS findings for follow-up validation/functional studies or fine-mapping of associated loci using linkage disequilibrium (LD) information (8).

Given the complex nature of gene expression regulation, and the critical roles of epigenetic systems including cytosine modifications (primarily DNA methylation at CpG dinucleotides) in gene regulation and in a broad range of biological processes, our team has undertaken a more comprehensive study of gene regulation by integrating cytosine modifications into the current HapMap resources (9). Specifically, we quantified cytosine modification levels of >480 000 CpG sites using the Illumina HumanMethylation450 BeadChip array (450K array) (10) in the European and African panels of the original HapMap samples that were previously used by our team to profile gene expression for eQTL mapping (4, 11). In addition, we mapped cytosine modification quantitative trait loci (mQTL) to investigate the genetic architecture of cytosine modifications, and found significant enrichment of mQTL for trait-associated GWAS loci in addition to eQTL (12, 13), highlighting the potential relevance of using mQTL as a functional annotation approach. Considering the complex relationships across CpGs, SNPs and gene expression, we may facilitate novel, integrative, and systematic functional genomic studies by providing an updated SCAN database with mQTL data. We describe here the updated SCAN database featuring the newly released mQTL mapping results.

## Database content update

SCAN has been expanded to integrate the newly released mQTL detected in 60 HapMap CEU (Caucasian residents from Utah, USA) and 73 YRI (Yoruba people from Ibadan, Nigeria) panels (13). The following subsections describe the mQTL data as well as new features that facilitate integrative analyses of these new data and the eQTL data featured in our previous version of SCAN (8). The original database architecture and the technical details about database implementation were described in our previous publication (8). We upgraded SCAN to the human genome reference build 19 and incorporated GENCODE (v20) gene-level annotations (14).

### Description of the mQTL data

#### Cytosine modification data

In total, 133 unrelated HapMap LCL samples (60 CEU and 73 YRI) were profiled for cytosine modification levels using the Illumina 450K array, which interrogates ∼480 000 CpGs across the human genome (10). Sample preparation and the 450K array profiling were described in Moen *et al.* (12). The raw and processed cytosine modification data including the summarized modification levels for each individual have been deposited into the NCBI Gene Expression Omnibus (Accession Number: GSE39672). Various filtering criteria were applied to the data to ensure that we had reliable cytosine modification profiles of these samples. To avoid potential probe cross-hybridization, which particularly could be an issue for a relatively degenerated target sequence after bisulfite treatment of genomic DNA, we re-aligned the 450K array probes to identify matched multiple genomic locations (12, 15). We also removed CpG probes containing common SNPs (minor allele frequency >0.01) (12) based on the dbSNP v135 database (16). Besides our internal controls using the same samples, comparing our 450K array data with the Encyclopedia of DNA Elements (ENCODE) Project (17) 450K array data for three same LCL samples (NA12891, NA12892 and NA19239) demonstrates the stability of cytosine modification profiles across experiments (r: 0.95-0.99) (12).

#### Detection of mQTL in the HapMap samples

Details on mQTL mapping were described in Zhang *et al.* (13). Briefly, 283 540 autosomal CpG sites that met our previously described criteria (e.g. calling rate>95%, not ambiguously mapped to multiple loci, not containing common SNPs) (12) were used in mQTL mapping. The M values, defined as the log_2_ ratio of the intensities of modified probe versus unmodified probe (18), were quantile normalized across all of the 133 samples, and adjusted for batch effect using COMBAT (19). Local scans of mQTL were then performed for SNPs <100 Kb away from the target CpG sites. Top principal components were regressed out to account for potential confounding variables and to achieve the greatest detection sensitivity in each population. Cytosine modification levels, i.e. M values, were regressed on SNP allele dosages within the CEU and YRI samples, separately.

#### Integration of mQTL data and other features of SCAN

**Figure 1.**
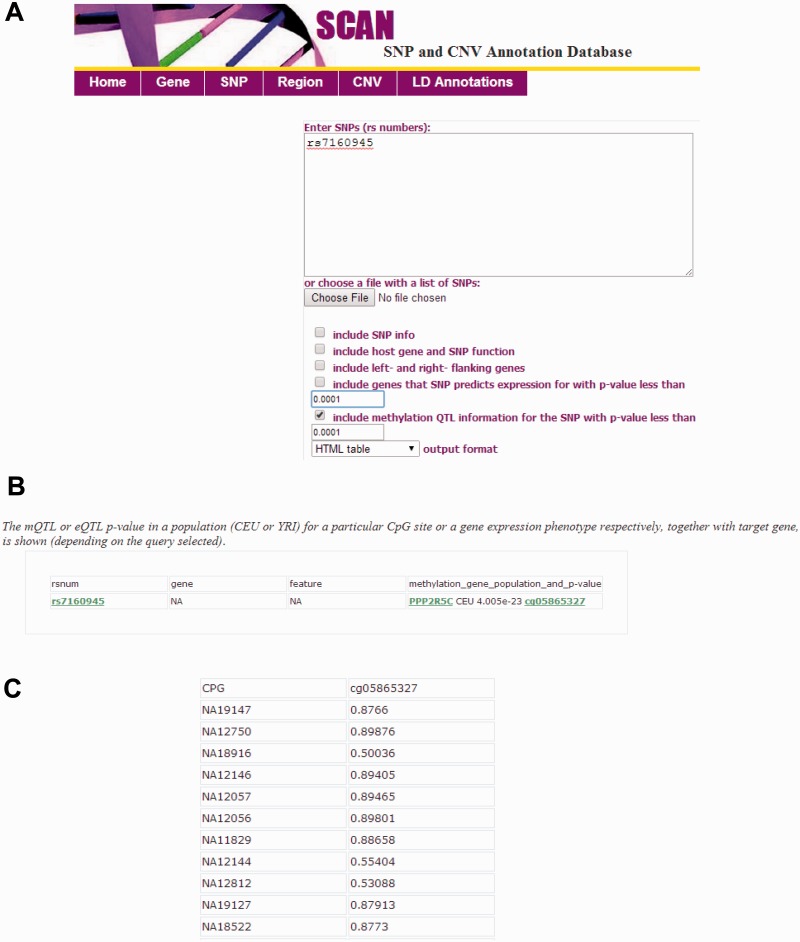
Interface of the updated SCAN database. (**A**) The SNP-focused interface is shown as an example. The updated SCAN supports both gene and SNP-focused queries. Users may specify significance level for the mQTL data (up to nominal *P* < 0.01). Batch queries are allowed. (**B**) An example for the output from a SNP-focused query is shown. The mQTL or eQTL *P* value in a population (CEU or YRI) for a particular CpG site or a gene expression phenotype respectively, together with target gene, is shown (depending on the query selected). CEU, Caucasian residents from Utah, USA; YRI, Yoruba people from Ibadan, Nigeria. (**C**) An example for the individual methylation level data (normalized β-values) accessed through the CpG identifier.

The SCAN database has been updated to integrate the mQTL mapped in the CEU and YRI samples. In total, 58 530 unique local mQTL for 5240 CpGs in the CEU and 43 412 mQTL for 7306 CpGs in the YRI (at nominal *P* < 0.01) samples were included in the updated database. The architecture of the underlying database is similar to the previous version of SCAN (8) for eQTL information. Briefly, the database has three tiers to allow extensibility, easy maintenance, and user-friendly database experience. The updated SCAN supports both gene and SNP-focused queries (batch queries allowed) and an option to specify significance level for the mQTL data. mQTL mapping results (i.e. *P* value and population) for significant CpG sites, together with target gene annotations and eQTL data are output from the SNP-query tool. Individual methylation levels (i.e. normalized β-values) can be displayed and downloaded from CpG identifiers (Figure 1). Relevant SNP, gene and genomic region information (e.g. genomic position, functional feature, LD information) are provided as the previous version of SCAN to provide genome context of these genetic variants. Examples of input and an online tutorial for the database are also provided at the SCAN website.

### Application examples

With the new mQTL data, the updated SCAN database allows annotation of genetic variants by not only eQTL but also mQTL from the HapMap CEU and YRI samples. We present two examples here to illustrate the potential applications of the new mQTL data and updated features of SCAN. Although two candidate genes are showcased here, the integrated mQTL and eQTL data provided by SCAN can support larger scale investigations through batch queries.

#### Case Study 1: Evaluation of mQTL associated with population-specific CpGs

The updated SCAN contains mQTL data generated from two global populations: persons of European (CEU) and African (YRI) descent. The following example shows how the integrated data may be used to identify genetic variants associated with population-specific CpG sites (12), which may underlie certain health disparities between ethnic groups.

Acute respiratory distress syndrome (ARDS) is a life-threatening condition with a substantial mortality rate (20). A significant disparity in mortality rate exists between patients of African descent and those of European descent, after controlling for access to health care and socioeconomic status (21), implying a possible genetic influence on clinical outcome. The underlying mechanisms of these health disparities in ARDS have been investigated to a great extent, but the precise processes remain indistinct. Prior studies have linked candidate genes involving endothelial and epithelial permeability to ARDS, particularly encoding myosin light chain kinase (*MYLK*), which has been thoroughly examined for its role in vascular endothelial cell barrier disruption and inflammatory responses (22). Exploring the potential functions of genetic variation in *MYLK* may help to interpret the observed health disparities in ARDS (23).

**Figure 2.**
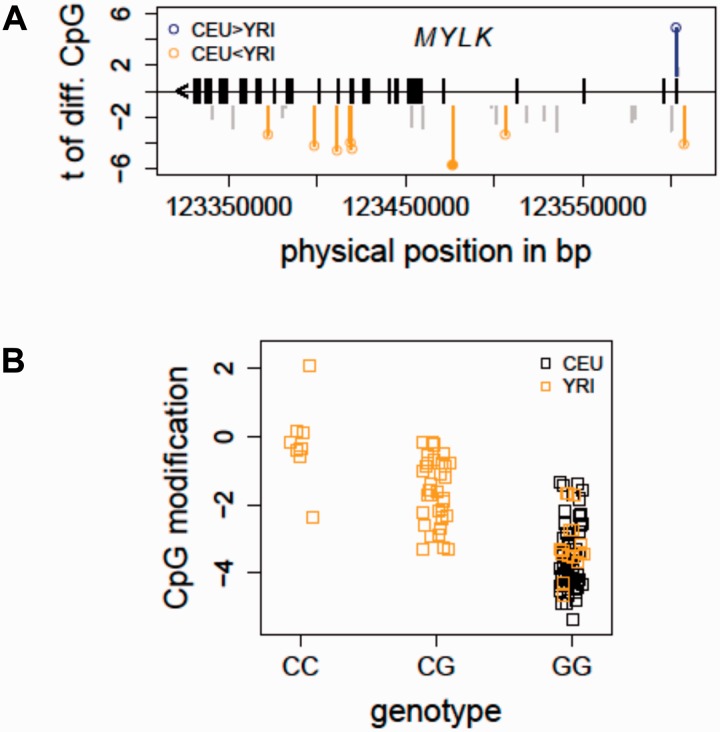
Detecting mQTL for population-specific CpGs - *MYLK* as an example. (**A**) The distribution of population-specific CpG sites in *MYLK* is shown. In total, 52 CpG sites in *MYLK* were profiled on the Illumina 450K array. At FDR<1%, eight CpG sites were characterized as differentially modified between the CEU and YRI samples. (**B**) An mQTL for a population-specific CpG in *MYLK* is shown. The allele C of an intronic SNP in *MYLK* (rs6438808) was found to be associated (*P* = 5.40e−10) with higher modification level of a gene body CpG (Illumina probe ID: cg12235788), explaining the higher modification levels in the YRI samples. CEU, Caucasian residents from Utah, USA; FDR, false discovery rate; MYLK, myosin light chain kinase; YRI, Yoruba people from Ibadan, Nigeria.

Taking advantage of the cytosine modification data profiled in the CEU and YRI samples (12), we evaluated if there were population-specific CpG sites located in the *MYLK* gene. At a false discovery rate (FDR) less than 1%, eight CpG sites (out of 52 total CpG sites in *MYLK* profiled on the 450K array) were characterized as differing between the two populations (Figure 2A), indicating a baseline cytosine modification variation pattern in *MYLK* between individuals of African and European ancestry. The integrated mQTL data in the updated SCAN were used to search for local genetic associations of these population-specific CpG sites. In particular, local SNPs were evaluated for association with all of the eight population-specific CpGs. Figure 2B shows an example in which the allele C (which has a frequency of 0.425 in YRI and is not present in CEU) of an intronic SNP in *MYLK* (rs6438808) was associated (*P* = 5.40e−10) with higher modification level of a gene body CpG (Illumina probe ID: cg12235788) in the YRI samples, suggesting that the underlying genetic variation may contribute to the observed population-specific cytosine modification variation in *MYLK*. Thus, findings in the HapMap samples provide some evidence for a relationship between the genetic and epigenetic variations in *MYLK*. Importantly, these association relationships could guide future functional and validation studies in patient cohorts to elucidate the observed ethnic disparities in ARDS (23).

#### Case study 2: integrative analysis of mQTL and eQTL

We show here an example of integrating mQTL and eQTL using the updated SCAN. Clinical studies and GWAS have demonstrated that the *MGMT* gene (encoding DNA repair protein *O*^6^-methylguanine-DNA methyltransferase) plays a role in sensitivity to temozolomide, an oral alkylating agent used for the treatment of brain tumor. For example, in a genome-wide pharmacogenomic study using 516 LCLs derived from a cohort of European descent, an eQTL (rs477692) in *MGMT* was identified to be associated with cytotoxic response to temozolomide (24). Previous studies also reported that promoter hypermethylation of *MGMT* could predict low expression levels of MGMT in gliomas, despite observed discordance between promoter methylation and protein levels. In 91 human glioblastoma samples from the Cancer Genome Atlas Project, we further observed significant variation in *MGMT* expression levels in patients with an unmethylated promoter, with higher levels of gene body cytosine modification correlating with higher gene expression levels (25).

**Figure 3.**
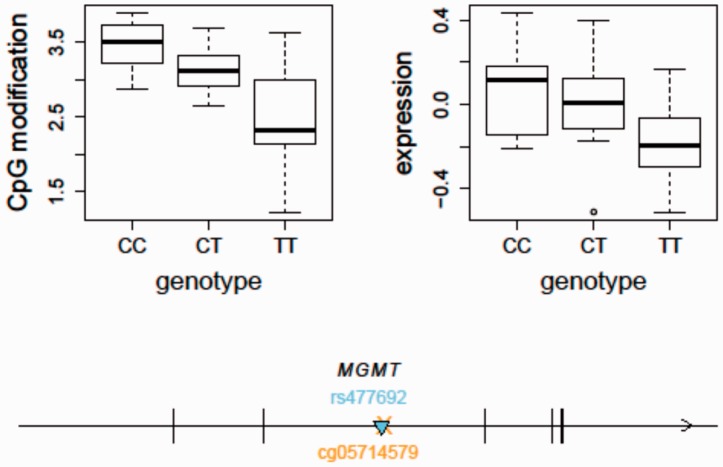
An integrative analysis of mQTL and eQTL - *MGMT* as an example. The temozolomide-associated SNP, rs477692, is an mQTL for *MGMT* gene body (Illumina probe ID: cg05714579) cytosine modification levels (*P* = 1.57e−7) and an eQTL for *MGMT* (*P* = 0.0001) expression in the CEU samples. The arrow indicates the transcription direction of MGMT. The triangle indicates the QTL position (*cis*-acting). The cross indicates the CpG location. MGMT, *O*^6^-methylguanine-DNA methyltransferase.

Using the updated SCAN, we found that the temozolomide-associated SNP, rs477692, is an mQTL for *MGMT* gene body (Illumina probe ID: cg05714579) cytosine modification levels (*P* = 1.57e−7) and an eQTL for *MGMT* (*P* = 0.0001) in the CEU samples based on our previously published gene expression data using the Affymetrix Human Exon 1.0ST Array (GSE9703) (Figure 3). In comparison, we did not observe this SNP to be an eQTL for *MGMT* in the YRI samples (*P* = 0.6). This may suggest a different set of genetic regulators of *MGMT* expression in the two populations although the difference in allele frequency at the SNP may also lead to a difference in statistical power. The integrative analysis of both eQTL and mQTL thus suggests that genetic variation (e.g. rs477692) may play a role in regulating *MGMT* expression through regulation of gene body CpG sites. Future clinical applications guided by these findings could improve the care of cancer patients receiving this therapy.

## Conclusions and future development

Understanding the genetic architectures of gene expression and cytosine modifications offer opportunities to assign functional annotations to genetic variants associated with human complex traits and phenotypes, such as those genetic loci identified in GWAS. Therefore, one of the primary applications of SCAN is in conducting follow-up analyses of the results from GWAS, including prioritizing GWAS findings for functional or validation studies. The updated SCAN has new features that allow queries for mQTL detected in the HapMap CEU and YRI samples together with relevant genome-context information. More importantly, the updated SCAN integrates mQTL into the existing eQTL resource from the same HapMap samples that have been widely used as a model of natural variation. Integration of mQTL and eQTL data will open up avenues to systematically characterize trait-associated loci from GWAS. Future developments may include integrating additional molecular phenotype trait loci measured in the same HapMap samples, such as protein-level QTL (pQTL) (26), as well as independent eQTL/mQTL data derived from other tissues such as findings from the Genotype-Tissue Expression (GTEx) Project (27). The SCAN database will be updated regularly to incorporate novel findings generated on these samples. Integration with other genomic resources including PACdb (28), a database for cell-based pharmacogenomics, may help to better elucidate the mechanisms underlying genetic variants implicated in drug response traits, which have been widely investigated using this model.
